# Study of Copper Electrodeposition at a Very Low Temperature near the Freezing Point of Electrolyte

**DOI:** 10.3390/mi13122225

**Published:** 2022-12-15

**Authors:** Yu Mo, Chunjian Shen, Di Zhu

**Affiliations:** College of Mechanical & Electrical Engineering, Nanjing University of Aeronautics and Astronautics, Nanjing 210001, China

**Keywords:** copper deposition, electrolyte temperature, cathodic overpotential, microstructure properties

## Abstract

At or above room temperature, metal electrodeposits often feature coarse grains, uneven microstructure and high roughness with abnormal bulges. In this study, copper electrodeposits with abnormal properties were prepared in a sulfate bath at a low temperature near the freezing point of the electrolyte. The results showed that the average grain size of the copper featured an “increase-decrease” trend while decreasing the temperature form 5 °C to −5 °C, yielding a trend from 0.25 μm to 1 μm and then to 0.6 μm. In the early stage, the temperature does not change the three-dimensional continuous nucleation mode of deposited copper. When the nucleus density reaches saturation, the polarization caused by overpotential will act on the respective nucleation and crystal growth process twice, and finally exhibit a completely different trend than that at room temperature. This study may provide insights for breakthroughs in material properties from a temperature perspective.

## 1. Introduction

Electrodeposition is a well-known additive manufacturing process. It utilizes the principle of electrochemical cathodic reduction to migrate metal cations to the cathode double layer under the action of an electric field, which are then reduced to atoms that accumulate on the cathode surface, finally forming metal parts with specific shapes and different properties. This method plays an important role in the manufacture of micro-nano structures, high aspect ratio holes and difficult-to-machine parts [1,2,3,4].

For a long time, efforts to understand the mechanism of electrodeposition and improve the properties of the electrodeposits have relied on studying the effects of various parameters on the electrodeposition. The cathode material [5], current density [6,7], cathode potential [8], additive concentration [9,10], and the pH value of the electrolyte [11] have a significant effect on the electrodeposition process. Jin et al. studied the effect of additive concentration on the microstructure of the deposited copper layer. With the change of additive concentration, the grain size and microstructure uniformity of the deposited product substantially changed [12]. Zhan et al. found the that the addition of additives improved the strength of electroformed copper microtubes [13]. Xue et al. studied the effect of current density on grain size [14]. The findings indicate that the fluctuation of the current density greatly affects the mechanical properties of the material, and columnar nano twinned copper with a hexagonal pyramid surface morphology is produced. Zhan et al. prepared nano twinned copper by replacing the DC power with pulse power [15]. This process prepared nano twinned copper with a regular pyramid shape. In addition, other auxiliary means can greatly change the organization and performance of copper deposition layer sample. Shen et al. studied the surface self-nanocrystallization electrodeposition, revealing the effect of mechanical assisted friction on the deposited copper layer [16].

Electrolyte temperature is also an important process parameter that affects the microstructure and macroscopic properties of deposited metals [17,18,19]. There are some interesting studies that found a better experimental performance after reducing the temperature parameters during the electrodeposition reaction. A. Mallik, et al. studied the effect of temperature and cavitation in the process of copper deposition, and found that the grain structure at low temperature is cleaner, finer and more regular than that at room temperature [20]. Xu et al. studied the effect of temperature on the electrodeposition process of Cu in CHCL-based deep eutectic solvents. As the temperature changes, the equilibrium potential moves in a positive direction, and the overpotential required for nucleation on the electrode surface decreases sharply. The author uses these factors to improve the three-dimensional nucleation of copper and to obtain denser deposits [21]. M. D. Groner et al. alternately exposed Al(CH_3_)_3_ and H_2_O to the low temperature electrodeposition state and found that the film density of Al_2_O_3_ was low at a lower deposition temperature, the film surface was very smooth, the roughness value was also low, and it had good electrical properties after measurement. Studies found that metals or metal composites deposited at low temperatures usually showed special or better properties [22]. However, when the temperature decreases to near the freezing point of an electrolyte, few reports have evaluated the influence of temperature change on the deposition performance of the copper layer, especially the nucleation and growth process of copper electrodeposition.

This paper studies copper electrodeposition at low temperature in the vicinity of the freezing point of the electrolyte. The results show that the grain size is not positively correlated with the electrolyte at ultra-low temperature. With the decrease of temperature, it decreases first, then increases and finally decreases. The change of temperature changes the electrolyte viscosity and the thickness of the diffusion layer on the cathode surface and reduces the diffusion coefficient of copper ions and deposition products to a certain extent, finally reducing the electrochemical reaction rate. Therefore, the variation of temperature changes the concentration polarization on the cathode surface, leading to the change of the cathode overpotential and breaking the balance between nucleation and growth. The copper electrodeposits exhibit a good comprehensive mechanical property. These results provide ideas for seeking a copper deposition process with better comprehensive mechanical properties.

## 2. Experimental Principles

Related studies have shown that reducing the temperature of the electrodeposition solution will effectively increase the overpotential of the cathode matrix, and a large overpotential may lead to a high nucleation rate, resulting in a large number of nuclei, so that it is greater than the grain growth rate and yields the corresponding fine grain structure [10].

To further study the effect of the electrolyte temperature on the microstructure and properties of copper deposited by direct current deposition, this work ensures that the process parameters, such as the circuit density, CuSO_4_ solution concentration and pH value are constant. Different temperatures (−5 °C~5 °C) are applied to the electrolyte by using a constant temperature circulation outer tank. Finally, layered Cu samples with different microstructures were prepared.

## 3. Experimental Device and Process

**Figure 1:**
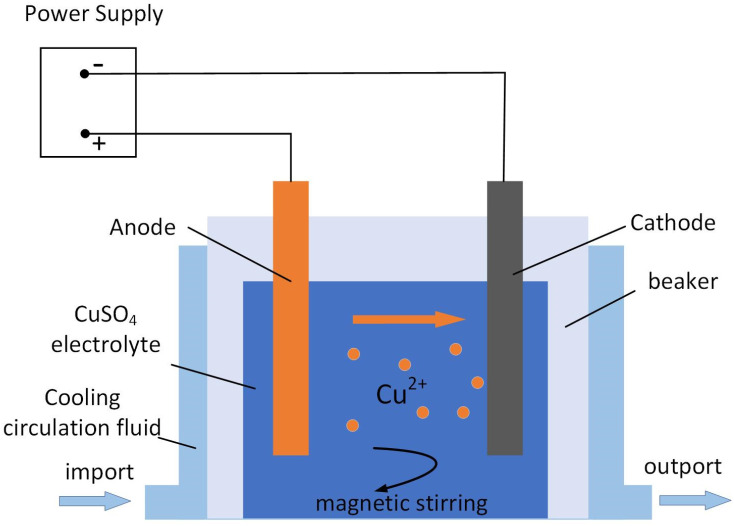
Schematic diagram of the experimental device and experimental reaction. There is a refrigerant circulating around the electrolyte. After the cathode and anode are powered the copper atoms on the anode lose electrons and become copper ions. The copper ions near the cathode are deposited on the cathode substrate.

In this experiment, an experimental platform was built with a beaker, cathode, anode, fixture, and low temperature circulation tank as components, and the direct current deposition experiment of electrodeposited copper was carried out at low temperature. The cathode consists of 304 stainless steel sheets, and it is implemented by 400–3000 mesh sandpaper grinding and cleaning, which is an indispensable process before use. The anode uses a phosphor copper plate and is wrapped in two layers of polyester cloth after ultrasonic cleaning, as shown in Figure 1. The experimental conditions are listed in Table 1. The test uses six temperatures for comparison (−5 °C, −3 °C, −1 °C, 1 °C, 3 °C, 5 °C). The experiment time of a single sample is 10 h, and the thickness of the copper layer is around 0.4 ± 0.08 mm. After the deposition is completed, the residual solution on the surface of the electrodeposition sample is washed with distilled water, dried, and stored with weighing paper. The obtained samples are cut and saved, one part for surface morphology observation and grain structure detection and the other part for mechanical property testing.

**Figure 2:**
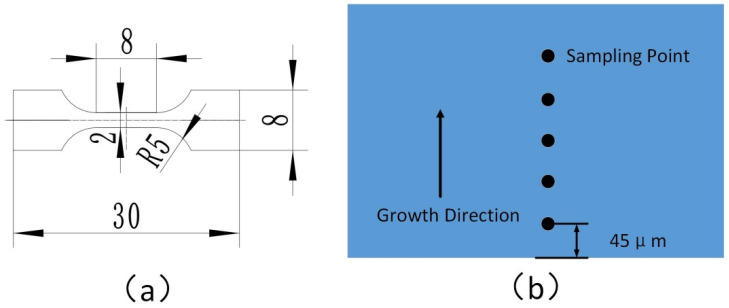
Standard tensile specimen pattern (**a**) and hardness testing schematic pattern (**b**). The sampling point will be marked every 45 μm along the grain growth direction on the cross section of the copper sample for hardness testing, as shown in (**b**).

In terms of microscopic morphology and microstructure analysis, the Oxford Nordly Max3 electron microscope is utilized to observe the grain size. Observation of the surface morphology and microstructure characteristics is conducted using a Tescan LYRA3 electron beam microscope. Observation of the metallographic structure is performed using a Zeiss 200 MAT optical microscope. Image Pro software is employed for grain size statistics. The crystal orientation information is analyzed using a Rigaku Smart Lab SE X-ray diffractometer. An atomic force microscope (Dimension Edge SPM; Bruker, Germany) is used to detect and analyze the surface roughness after machining. Some samples used for mechanical property testing need to be machined into standard tensile samples by wire cutting with electric discharge, then polished with 2000 mesh sandpaper and electrolyzed with phosphoric acid aqueous solution to remove the oxide and stress layers on the surface of the copper coating. The tensile property is tested by a universal tensile specimen machine (CSS-2202). The strain rate during tension is 6.7 × 10^−4^ s^−1^, and the elongation is measured after fracture of an 8mm gauge length section by scribing. The cross section of the copper casting layer surface is tested by a microhardness tester (HXS-1000A). The loading is 0.5 N, the holding time is 15 s, and sampling is performed every 45 μm of the base surface. The hardness of the sampling points on each sample is counted, and the average value is taken as the overall hardness of the sample, as shown in Figure 2.

## 4. Results and Discussion

### 4.1. Microstructures

**Figure 3:**
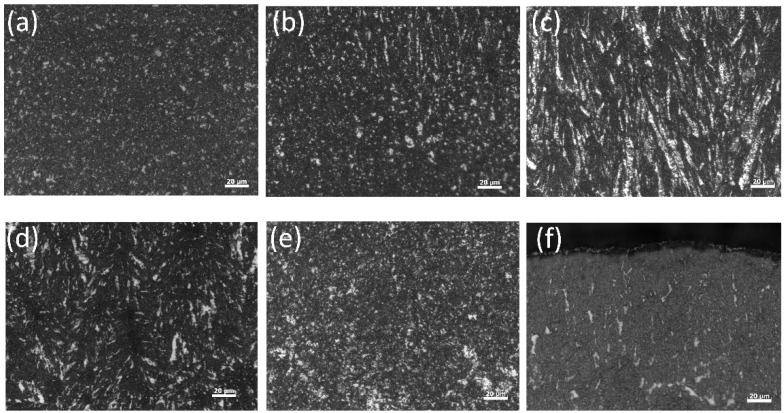
Metallographic organization diagram of copper deposits: (**a**) −5 °C, (**b**) −3 °C, (**c**) −1 °C, (**d**) 1 °C, (**e**) 3 °C, (**f**) 5 °C. The distributions of the metallographic structure in (**c**,**d**) are uneven, and the size is obviously larger than that at other temperatures.

Figure 3 shows the metallographic organization of the electrodeposited copper layer at different temperatures, revealing the metallurgical phase changing trend of the sample. Figure 3c,d have a large grain size, including a large number of columnar crystals. Figure 3a,f have a small grain size, showing a trend of high intermediate temperature and low temperature on both sides. The reason is that the change in the electrolyte temperature affects the nucleation and growth process of metal deposition [17,18,19]. At the beginning, the reduction and nucleation of metal atoms did not have the limitation of epitaxial growth and allow the growth to proceed freely. A dense equiaxed crystal was formed near the cathode substrate. Then, under the influence of epitaxial growth, the crystal nucleus grew along the direction perpendicular to the cathode substrate. The closest layer of the crystal nucleus near the cathode gradually grew outward in the form of columnar crystals.

**Figure 4:**
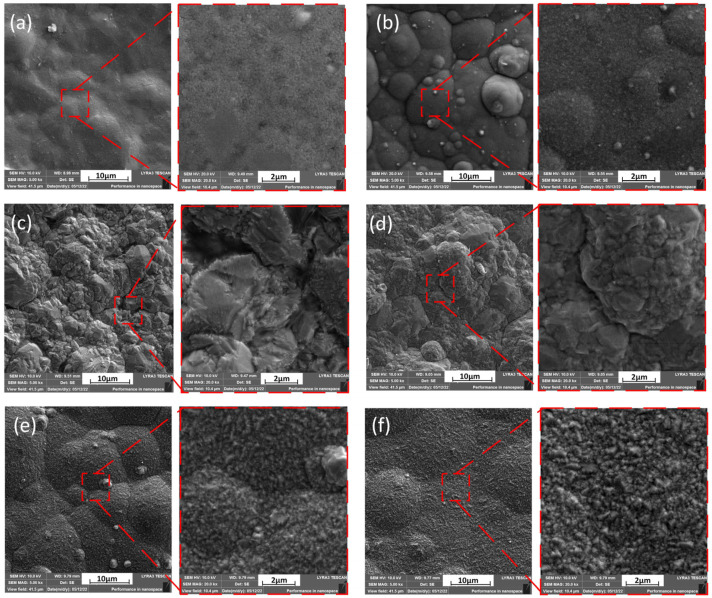
Microstructures of the sample surface and local enlargements of their feature areas at different low temperatures ranging from −5 °C to 5 °C. (**a**) −5 °C, (**b**) −3 °C, (**c**) −1 °C, (**d**) 1 °C, (**e**) 3 °C, (**f**) 5 °C. (**e**,**f**) show the electrodeposited copper sample with the smallest grain size and the most delicate structure, followed by (**a**,**b**). The electrodeposited copper layer in (**c**,**d**) has the roughest surface and the largest grain size.

**Figure 5:**
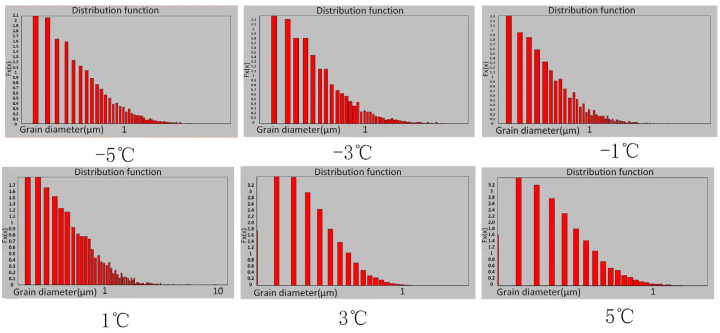
Distribution diagram of the corresponding grain size at different temperatures from −5 °C to 5 °C. Compared to other temperatures, the grain size is smallest and most concentrated at 5 °C and 3 °C.

Figure 4 shows the Scanning Electron Microscopy (SEM) images of the electrodeposited copper layer, which characterizes the microstructure and terrain morphology of the surface. If these six temperatures are classified, it is obvious from the diagram that the grain sizes at 5 °C and 3 °C maintain the same order of magnitude, the grain sizes at −3 °C and −5 °C also follow the same order of magnitude, and the grain sizes at 1 °C and −1 °C have approximately the same order of magnitude. When the temperature was reduced from 3 °C to 1 °C, a leap-forward change was observed on the surface of the sample under the same magnification, which was significantly rougher than that in other images. The surface structure of the copper layer exhibited a fine appearance without large bulges and gullies at 5 °C, as shown in Figure 4f, then a slight bulge at 3 °C, as shown in Figure 4e, and large gullies, uneven particles and a rock structure at 0 °C. The sharp ravines and rock structure on the surface disappeared, and revealed a smooth, convex surface at −3 °C, and then the surface became smooth and the structure was symmetrical at −5 °C. Through the observation of backscattered electron microscopy, we obtained the distribution map of the grain size at different temperatures, as shown in Figure 5. The grain diameter at 5 °C and 3 °C are almost all less than 1 μm. After the temperature reaches 1 °C, some grain sizes increase to more than 1 μm, revealing the phenomenon of mixed grain species.

The growth process of deposited metal includes the following steps: the diffusion of solution metal ions to the vicinity of cathode, the electrochemical process of reduction through the interface between the cathode metal and the solution, and the electrocrystallization process of nucleation and growth of the reduced metal atoms on the cathode surface. Decreasing the temperature increases the electrolyte viscosity [19,20,21,22,23] and cathode surface diffusion layer thickness [5]. At the same time, it will reduce the diffusion coefficient of copper ions and copper atoms and the electrochemical reaction rate constant, which will decrease the adsorption of copper ions [24], the reduction [23,24,25], the surface diffusion of copper atoms [25,26], and other electrochemical processes of copper deposition. As a result, this behavior will increase the concentration polarization phenomenon, the cathode overpotential, and the driving force in the nucleation process, reduce the nucleation radius, and almost increase the nucleation probability. At this time, the formation rate of the crystal nucleus in the electrodeposition process is greater than the growth rate of the crystal nucleus; that is, the nucleation rate is mainly promoted, which is beneficial in obtaining a copper layer with a smaller grain size.

When the temperature is approximately 0 °C, according to the theory that posits that the continuous nucleation will eventually reach the saturation value in a certain area proposed by Depestel [27] and Michev [28], the findings indicate that the active sites on the surface of the substrate are limited. Although copper nucleation and crystal growth occurred at different temperatures, the change in temperature did not affect the three-dimensional continuous nucleation pattern of the copper. Before the nucleation density reached saturation, the decrease in temperature promoted both nucleation and crystal growth rates, but after the saturation, nucleation density was reached in the later stages of electrodeposition, and the promotion of crystal growth was dominant [27,28,29].

Since energy always flows preferentially to the site with a greater energy gradient, some of the larger crystals rapidly absorb the overpotential spillover energy for growth and merging because of easier access to energy. Contrary to this, other nuclei obtain less energy and grow more slowly, thus ultimately exhibiting the phenomenon of the mixing of small and large grains.

When the temperature continues to decrease and the concentration polarization further increases gradually, the overpotential can again grow beyond the absorption range of larger crystals. Then excess energy will be replenished to the smaller crystals to help them grow, and when they grow to a certain extent, it will weaken the growth of the previous larger crystals. Ultimately, the tissue starts to become uniform, yielding a small overall trend [30,31].

**Figure 6:**
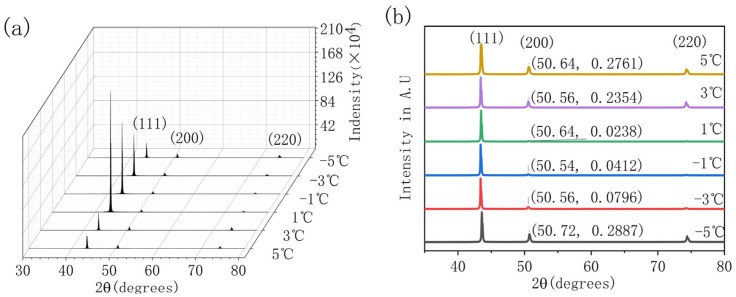
The XRD waterfall pattern (**a**) and y-axis offset stack pattern (**b**) of copper samples deposited at low temperatures from −5 °C to 5 °C. The intensity ratio of (111) peak to (200) peak is shown in (**b**).

Figure 6a shows the surface X-ray diffraction (XRD) pattern of the electrodeposited copper layer, which characterizes the change of crystal orientation. Upon observation, all samples have a body-centered cubic structure, and the crystal planes corresponding to each diffraction peak are (111), (200), and (220). No other foreign phase was observed in all samples, and the (111) peak represented a preferential orientation. At different temperatures, the peak intensities of (200) and (220) peaks are weak and approximately the same, while the (111) peaks are significantly different. In the process of gradually changing the temperature from 5 °C to −5 °C, the peak intensity of the (111) peak increases very fast and then decreases gradually. In particular, the peak intensity has a sharp jump in the process of changing from 3 °C to 1 °C, such that the diffraction peak intensity almost reaches the highest point at 1 °C.

Figure 6b is the image obtained after normalization according to the maximum diffraction peak of a single sample. The relative ratio of the diffraction peak of the (220) crystal plane to the (111) crystal plane decreases from 27.61% at 5 °C to 2.38% at 1 °C and then increases from 2.38% at 1 °C to 7.96% at −3 °C. After a small increase, the final jump increases to 28.87% at −5 °C. Relevant studies have shown that under normal circumstances, in a specific temperature range, the larger the grain size is, the higher the corresponding diffraction peak intensity [32]. According to the above-mentioned microstructure of the Cu sample, it can be seen that when the temperature is reduced to 1 °C, the density of the crystal nucleus reaches the saturation density of the crystal nucleus at this temperature, the residual energy caused by the increase of the overpotential promotes the growth and merging of the crystal, and then finally lets the grain size increase sharply in a disorderly fashion. This may result in the corresponding diffraction peak suddenly increasing and the half-peak width becoming narrower. Subsequently, an increasing number of crystal nuclei develop in the direction of crystal growth after continuing to reduce the temperature and increase the overpotential. Therefore, it compresses the space for original large crystal growth, making the overall grain size smaller, and the diffraction peak gradually decreases.

These findings serve to substantiate the conjecture that the change in temperature leads to the change of surface grain structure, surface diffraction density and orientation index. Relevant research also shows that the (111) peak of the deposited copper layer has the highest elastic modulus, which may be helpful to the performance of the material [33].

### 4.2. Mechanical Properties

**Figure 7:**
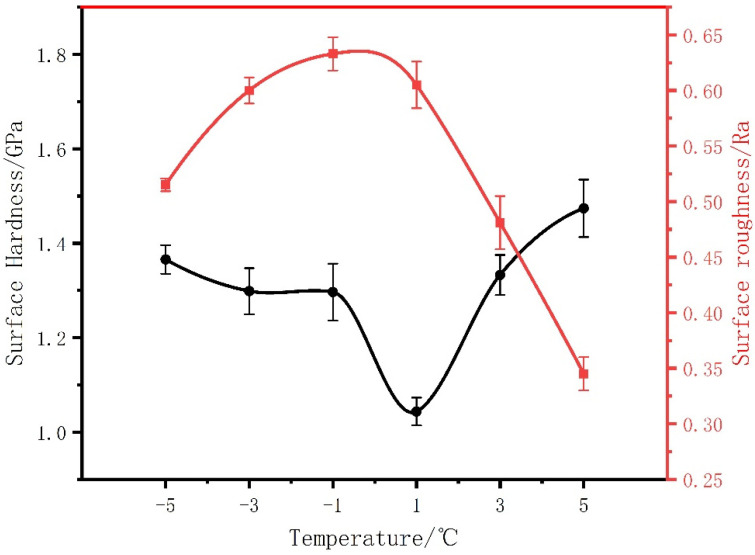
Surface hardness and roughness of the copper deposits produced at different temperatures from −5 °C to 5 °C. Among them, the surface hardness of the copper deposit layer fluctuates most obviously at 1 °C, and is as low as 1 GPa.

Figure 7 shows the surface roughness of copper samples at different temperatures. The figure shows that the surface relative roughness reaches the highest value, and the maximum data among them are close to 0.65 Ra. Theoretically, the finer the grain structure of the copper deposited layer is, the lower the corresponding surface roughness value. Figure 7 also shows the surface hardness curve of the electrodeposited copper layer. The figure illustrates that the surface hardness of the copper deposition layer at a low temperature is slightly lower than that at a normal temperature, but in the same grade range, the smaller the grains are, the higher the corresponding surface hardness. It is obvious that the surface hardness of the copper layer decreased precipitously at 1 °C, approaching 1 GPa; for comparison, the maximum surface hardness is 1.45 GPa at 5 °C.

The grain size of the sample is examined by an Oxford Nordly Max3 electron microscope, as indicated in the previous section and shown in Figure 5. There is an interesting phenomenon that the average grain diameter increases several times when the electrolyte temperature reached 1 °C, and this indicates a large jump in the grain size. As discussed in the case of the nucleation density reaching the saturation density, lowering the temperature induces a continued increase in the overpotential and intensification of the concentration difference polarization. The energy from the overflow will be partially converted into energy to promote the growth of small grains combined as large nuclei, and in the process of mixed growth of the large and small grains, the phenomenon of a large grain becoming larger and a small grain becoming smaller is evident. In summary, this uneven distribution breaks the original grain boundary layer structure so that it is not as sufficiently solid as it was previously. Therefore, a likely explanation is that a combination of these factors induces the mechanical properties of the sample as it changes abruptly when the temperature decreases to 1 °C.

**Figure 8:**
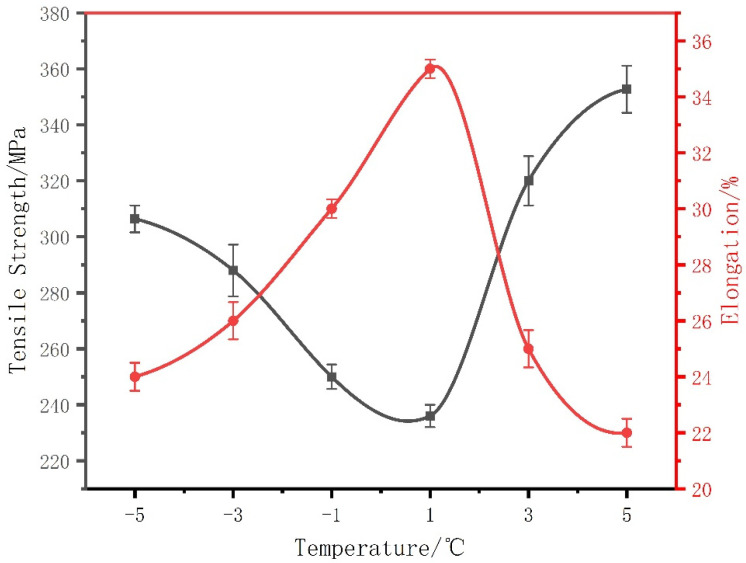
Tensile strength and elongation of copper deposits produced at different temperatures from −5 °C to 5 °C. The tensile strength of the copper deposit is the highest at 5 °C, and the elongation of the copper deposit is the highest at 1 °C.

The change of tensile strength with temperature is shown in Figure 8. The minimum tensile strength is 240 MPa and the maximum tensile strength is 350 MPa, which is increased by 37.5%. The elongation reaches a maximum of 35% at 1 °C and a minimum of 22% at 5 °C, as shown in Figure 8. The small grain size increases the tensile strength of the overall material at 5 °C, but this greatly reduces its original elongation.

In addition to the above reasons for the influence of the grain size, the interaction between dislocation and twin boundaries plays a crucial role in the mechanical properties of the material [34]. On the one hand, dislocations can be piled up on the twin boundary, which can effectively hinder the movement of dislocations and play a role in strengthening the material. When the deformation stress is high enough, dislocations react with the twin boundary and pass through the twin boundary. On the other hand, the twin interface is also a slip surface. The twin boundary surface facilitates dislocations to not only move on its surface but also provides the coherent twin interface as space for the storage of dislocations during deformation, thus effectively improving the plasticity and work hardening ability of the material. Studies have shown that temperature changes have a significant effect on the energy of metal dislocation, which may greatly affect the dislocation and slip between the metal interface layers [35]. Such temperature dependence of metal effects warrants special attention. Between the two peaks, the deposited copper layer at a temperature of ±3 °C has good comprehensive mechanical properties, which provides a certain reference value for the search for structural materials with excellent comprehensive mechanical properties.

**Figure 9:**
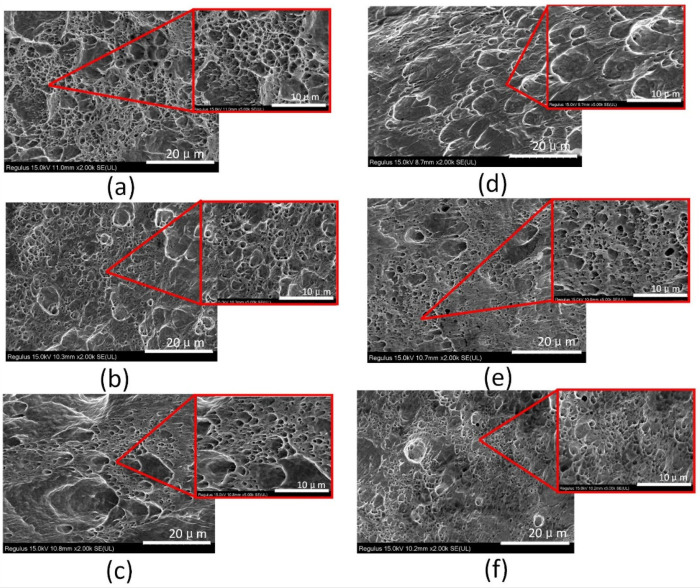
Fracture surface morphology of copper deposits with temperatures from −5 °C to 5 °C after strength testing: (**a**) −5 °C, (**b**) −3 °C, (**c**) −1 °C, (**d**) 1 °C, (**e**) 3 °C, (**f**) 5 °C. The dimple shape, size, depth, width and uniformity are quite different at various temperatures.

Figure 9 shows the fracture surface morphology of copper deposits after strength testing. The fracture surface morphology can reflect the mechanical properties of copper deposits. Figure 8 and Figure 9 illustrate that the morphological changes in the fracture surface are consistent with the variation trends of the tensile strength and elongation ratio of the copper deposits. As shown in Figure 9, the mode of tensile fracture was a dimple fracture. The copper deposits have different fracture morphologies at different temperatures. After careful observation, they have great differences in the shape, quantity, density, size, depth, width and distribution of the dimples. However, the mechanical properties of the deposits determined the number, dimension and distribution of dimples. An examination of the dimples in all the samples indicates that the greatest number of dimples in the microstructure occurred at 3 °C and 5 °C. Moreover, the dimples were shallow and small at this temperature. This implies a high resistance to deformation and a high tensile strength. The most homogeneous distribution of dimples in the microstructure was obtained at the electrolyte temperature of approximately 5 °C. Furthermore, the dimples achieved their maximum depth and width at ±1 °C, as well as exhibiting a high degree of deformation and thus the highest elongation ratio.

## 5. Conclusions

In this paper, the effects of temperature on metal ion reduction, nucleation formation and growth during the formation of deposited copper were studied at the limit temperature near the freezing point of the material. The findings show that the change in temperature will not change the three-dimensional continuous nucleation mode of deposited copper in the process of low temperature electrodeposition. When the density of the crystal nucleus reaches saturation, the polarization caused by overpotential acts on the respective nucleation and crystal growth process twice and finally produces a completely different trend from the normal temperature state. Furthermore, without any additives, the grain diameter size can be greatly refined by only a large decrease in the temperature, resulting in a smooth surface, low roughness, and balanced elongation and tensile strength. This provides a new idea for obtaining materials with high comprehensive mechanical properties by decreasing the temperature.

Overall, the change in the temperature will affect both the size of the overpotential and the relationship between nucleation and growth, ultimately changing the morphology and properties of the material. Therefore, the low temperature process has introduced new highlights to electrodeposition manufacturing.

## Figures and Tables

**Table 1:** Experimental Conditions.

Experimental Factors	Numerical Value
CuSO_4_·5H_2_O concentration	220 g/L
H_2_SO_4_ concentration	60 g/L
Cathode current density	4 A/dm^2^
Electrolyte temperature	(−5~5) ± 0.1 °C

## Data Availability

Not Statement.
